# The m^6^A RNA Modification Modulates Gene Expression and Fibrosis-Related Pathways in Hypertrophic Scar

**DOI:** 10.3389/fcell.2021.748703

**Published:** 2021-11-15

**Authors:** Si-Yu Liu, Jun-Jie Wu, Zhong-hua Chen, Ming-Li Zou, Ying-ying Teng, Kai-Wen Zhang, Yue-Yue Li, Dang-yang Guo, Feng-Lai Yuan

**Affiliations:** ^1^Department of Medicine, Institute of Integrated Traditional Chinese and Western Medicine, Wuxi Hospital of Integrated Traditional Chinese and Western Medicine, Nanjing University of Traditional Chinese Medicine, Wuxi, China; ^2^Institute of Integrated Chinese and Western Medicine, The Hospital Affiliated to Jiangnan University, Wuxi, China; ^3^Department of Medicine, The Nantong University, Nantong, China; ^4^The Hospital Affiliated to Jiangnan University, Wuxi, China

**Keywords:** hypertrophic scar, m^6^A sequencing, modification patterns, N6-methyladenosine, MeRIP-seq

## Abstract

**Purpose:** To systematically analyze the overall m^6^A modification pattern in hyperplastic scars (HS).

**Methods:** The m^6^A modification patterns in HS and normal skin (NS) tissues were described by m^6^A sequencing and RNA sequencing, and subsequently bioinformatics analysis was performed. The m^6^A-related RNA was immunoprecipitated and verified by real-time quantitative PCR.

**Results:** The appearance of 14,791 new m^6^A peaks in the HS sample was accompanied by the disappearance of 7,835 peaks. The unique m^6^A-related genes in HS were thus associated with fibrosis-related pathways. We identified the differentially expressed mRNA transcripts in HS samples with hyper-methylated or hypo-methylated m^6^A peaks.

**Conclusion:** This study is the first to map the m^6^A transcriptome of human HS, which may help clarify the possible mechanism of m^6^A-mediated gene expression regulation.

## Introduction

Hypertrophic scars (HS) normally occur after burns and trauma. The pathological features of hypertrophic scars include abnormal inflammation, excessive proliferation and differentiation of fibroblasts, increased angiogenesis, and excessive deposition of extracellular matrix (ECM) (Zhu et al., 2020; Zhang et al., 2021). The clinical manifestations are accompanied by itching (Lee et al., 2004), pain (Zhuang et al., 2021), infection (Zhu et al., 2007), and even dysfunction (Bijlard et al., 2017). When HS occur on the face, joints, or other important parts of the body, they will negatively impact the patient’s appearance and seriously affect physical and mental health (Gurtner et al., 2008; Eming et al., 2014). Current treatment methods for HS have certain limitations. It is difficult to achieve a complete cure by traditional surgery, radiotherapy, and hormone therapy, particularly in areas where HS are prone to recurrence (Jaloux et al., 2017). Therefore, scar repair has always been a difficult and hot spot in the field of wound repair and plastic surgery. At present, the etiology and molecular mechanisms that lead to the continuous over-synthesis of collagen in HS are still unclear. Studies have shown that regulatory factors including epigenetic DNA methyltransferase, non-coding RNA, and histones are dysregulated in skin fibrosis (Mann and Mann, 2013; Hardy and Mann, 2016; Lee et al., 2017). Evidence accumulated in preclinical studies and first proof-of-concept studies in patients with fibrosis indicates that targeted abnormal epigenetic modifications may provide potential for the treatment of skin fibrosis (Noda et al., 2014; Zeybel et al., 2017; Shin et al., 2019). The regulation of the apparent modification of m^6^A is one of the important foundations for cell fate changes and decisions, and it is also a new research hotspot in life sciences. Therefore, whether m^6^A can be used as a new target and mechanism for fibrotic diseases and provide new treatment method for skin fibrosis remains to be seen.

N6-methyladenosine (N6-methyladenosine, m^6^A) is the most significant modification of poly-adenylated mRNAs and long non-coding RNAs in higher eukaryotes, which was first reported during the early 1970s (Wei et al., 1975). The m^6^A is the most common mRNA modification in mammals (Zhang et al., 2017). In recent years, greater attention has again been paid to the fact that it plays an important role in many aspects of RNA metabolism. The m^6^A modification targets and regulates the stability (Wang et al., 2014), localization (Wang et al., 2014), transport, and translation of mRNA after transcription (Wang et al., 2015), thereby affecting various biological processes, including embryonic development, stem cell self-renewal, DNA damage response, and primary miRNA processing (Wang et al., 2014; Weng et al., 2019; Liu et al., 2021). The m^6^A modification is catalyzed by the m^6^A methyltransferases (termed m^6^A writers), including methyltransferase-like 3 and 14 (METTL3 and METTL14), and the co-factor, Wilm’s tumor 1 associated protein (WTAP) (Fu et al., 2014). In contrast, m^6^A demethylases, including fat mass and obesity-associated protein (FTO) and AlkB homolog 5 (ALKBH5) (also known as m^6^A erasers), can remove methyl radicals from RNA, maintaining the m^6^A modification in dynamic equilibrium (Li et al., 2019). Another group of m^6^A binding proteins (such as the YT521-B homology domain family YTHDF1/2/3) act as m^6^A readers to mediate specific functions of methylated mRNA transcripts (Fu et al., 2014; Deng et al., 2018). The m^6^A modification is a dynamic process; due to its tissue specificity, it has spatiotemporal characteristics, and it responds to internal and external signals. Indeed, ever increasing evidence shows that m^6^A modification is not only associated with normal biological processes but also with the occurrence and development of different types of diseases. In 2012, two independent studies reported for the first time the m^6^A RNA methylome in the mammalian genome, using the m^6^A-RNA immune-deposition method followed by high-throughput sequencing (MeRIP-seq). However, the distribution of the m^6^A transcriptome in most diseases is largely unknown. Skin has a high m^6^A content. Studies have shown that m^6^A can affect the selection of skin phenotype transformation (Xi et al., 2020), but the role of this regulatory mechanism remains unclear (Dominissini et al., 2012; Meyer et al., 2012). To date, the RNA m^6^A methylation profile of HS has not been determined. We report here for the first time the m^6^A profile within the transcriptome in HS samples and adjacent NS, showing a highly diverse m^6^A modification pattern between these two different types of skin. Abnormal m^6^A RNA modification in HS has been shown to regulate the expression of fibrosis-related genes and pathways. The present study will help in the further investigation of the potential role of m^6^A modification in HS pathogenesis.

## Materials and Methods

### Patients and Samples

This study was approved by the Ethics Committee of the Affiliated Hospital of Jiangnan University, and written informed consent was obtained from all the participants. All procedures performed in the research involving human participants complied with ethical committee. HS tissue and adjacent full-thickness normal skin (NS) tissue were collected during plastic surgery (Table 1). Following sample collection, this was immediately transferred into a 1.5-ml RNase-free centrifuge tube, rapidly frozen in liquid nitrogen, and stored at −80°C until RNA separation. Three pairs of scars for the HS group and normal skin samples for the NS groups were selected for MeRIP and RNA sequencing, and the remaining samples were stored at −80°C until used.

**TABLE 1:** Clinical characteristics of patients.

Hospital ID	Age (y)	Sex	Type of wound
0000726681	29	Female	Hypertrophic scar
0000726621	25	Male	Hypertrophic scar
0000726817	27	Male	Hypertrophic scar
0000726681	29	Female	normal skin
0000726621	25	Male	normal skin
0000726817	27	Male	normal skin

### High-Throughput m^6^A and mRNA Sequencing

m^6^A RNA-Seq service was provided by Cloudseq Biotech Inc. (Shanghai, China). Briefly, m^6^A RNA immunoprecipitation was performed with the GenSeq m^6^A-MeRIP Kit (GenSeq Inc., China) by following the manufacturer’s instructions. Both the input samples without immunoprecipitation and the m^6^A IP samples were used for RNA-seq library generation with NEBNext Ultra II Directional RNA Library Prep Kit (New England Biolabs, Inc., United States). The library quality was evaluated with BioAnalyzer 2100 system (Agilent Technologies, Inc., United States). Library sequencing was performed on an Illumina Hiseq instrument with 150 bp paired-end reads. RNA high-throughput sequencing was performed by Cloud-Seq Biotech (Shanghai, China). Briefly, total RNA was used for removing the rRNAs with NEBNext rRNA Depletion Kit (New England Biolabs, Inc., MA, United States) following the manufacturer’s instructions. RNA libraries were constructed by using NEBNext Ultra II Directional RNA Library Prep Kit (New England Biolabs) according to the manufacturer’s instructions. Libraries were controlled for quality and quantified using the BioAnalyzer 2100 system (Agilent Technologies, Inc., United States). Library sequencing was performed on an Illumina Hiseq instrument with 150 bp paired-end reads. Raw data of RNA-seq and m^6^A-seq have been uploaded to GEO database (accession number GSE181540)^1^.

### Sequencing Data Analysis

Briefly, paired-end reads were harvested from an Illumina HiSeq 4000 sequencer and were quality controlled by Q30. After 3′ adaptor-trimming, low quality reads were removed by cutadapt software (v1.9.3). First, clean reads of all libraries were aligned to the reference genome (HG19) by Hisat2 software (v2.0.4). Methylated sites on RNAs (peaks) were identified by MACS software. Differentially methylated sites were identified by diffReps. These peaks identified by both software overlapping with exons of mRNA were figured out and chosen by home-made scripts. Identified m^6^A peaks were subjected to motif enrichment analysis by HOMER (Heinz et al., 2010), while the compared reads can be visualized in the Integrative Genomics Viewer (IGV) to visually show the expression of mRNA (Thorvaldsdóttir et al., 2013). GO and pathway enrichment analysis were performed by the differentially methylated protein coding genes.

### RNA Extraction and Quantitative Real-Time PCR

TRIzol Reagent (Life Technologies, CA, United States) was used to isolate total RNA, which was used to synthesize complementary DNA with the iScript cDNA Synthesis Kit (Takara, Liaoning, China). Real-time PCR was subsequently performed using an SYBR premix Ex Taq (Takara) and the ABI 7500 Sequence Detection System (Thermo Fisher Scientific, MA, United States). All procedures were performed according to the manufacturer’s protocols.

### Gene-Specific m^6^A Quantitative Real-Time PCR Validation

Four genes with differentially methylated sites according to the m^6^A-seq were tested by reverse transcription (RT)-Quantitative Real-Time PCR (qPCR). A portion of fragmented RNA was saved as the input control. The remaining RNA was incubated with anti-m^6^A antibody-coupled beads. The m^6^A-containing RNAs were immunoprecipitated and eluted from the beads.

Both input control and m^6^A-IP samples were subjected to RT-qPCR with gene-specific primers. The gene-specific qPCR primers could be seen in Table 2.

**TABLE 2:** Sequences of primers used for qRT-PCR analysis of mRNA levels.

Name	Sense	Antisense
GAPDH	AATCCCATCACCATCTTCCA	TGGACTCCACGACGTACTCA
COL11A1	TGGTCAAATTGGCCCAAGA	CTTTTCTCCTGCTTGACCTGA
COL8A1	AGCTGTTGTGAAGGCAGAGC	AGACCAATGTCCTTGCTGGT
AR	CCACCTCCAAGATCCCTACA	CAGAAGATGACAGAGGCCAGA
CYP1A1	ACTCACCTGTGAGGGACTGG	TGCCAACTTGTTTGGAGATG

### RNA m^6^A Quantification

Total RNA was extracted from cells using TRIzol reagent (Life Technologies, CA, United States). The m^6^A RNA Methylation Assay Kit (ab185912; Abcam, United Kingdom) was used to detect the m^6^A content in the total RNA. In brief, 2 μl negative control, 2 μl diluted positive control, and 200 ng sample RNA were added to a 96-well plate. Subsequently, 50 μl diluted capture antibody, detection antibody, and diluted enhancer solution were added into each well. Following the addition of 100 μl diluted developer solution to each well, stop solution was added, and the signaling was detected within 2 to 10 min using a microplate reader at 450 nm. A simple calculation of the percentage of m^6^A in total RNA was performed using the following formula: m^6^A% = [(sample OD - NC OD) ÷ S]/[(PC OD - NC OD) ÷ P] × 100%. S is the amount of sample RNA (ng), P is the amount of positive control (PC, ng), and NC is the negative control (ng).

### Statistical Analysis

Data from three or more independent experiments were presented as the mean ± SD. Statistical analysis was performed using SPSS 22.0 and GraphPad Prism 5.0 software. Paired Student’s *t*-tests were performed between HS and adjacent NS samples. One-way ANOVA was used to access the differences among three or more groups. Differences with *p* < 0.05 were defined as the threshold for significance.

## Results

### Transcriptome-Wide m^6^A-Seq Revealed Global m^6^A Modification Patterns in Hypertrophic Scar

Selected HS tissues and adjacent NS tissues from three patients were obtained for transcriptome m^6^A sequencing (m^6^A-seq) and RNA sequencing (RNA-seq) analysis methylated RNA immunoprecipitation sequencing IP or input in HS and NS. From these samples 37,973,160–43,157,012 original reads were obtained. After filtering out low-quality data, more than 33 million high-quality reads in each sample were mapped to the *Gallus gallus* reference genome (Gallus^.^gallus-5.0). The clean reads of above 82% obtained from all samples were uniquely mapped to the reference genome (Supplementary Table 1), which allowed us to effectively identify the m^6^A peak by analyzing paired MeRIP and input libraries.

**FIGURE 1:**
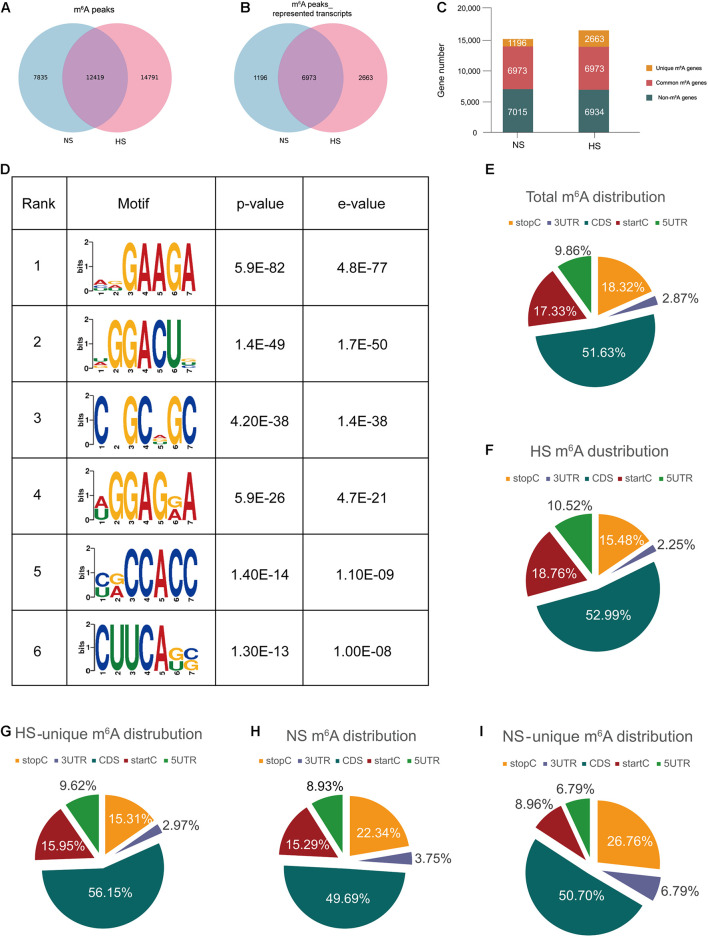
Transcriptome-wide m^6^A-seq and analysis of m^6^A peaks. **(A)** Identification of m^6^A peaks by model-based analysis of the ChIP-seq algorithm. The numbers of hypertrophic scar-unique, normal skin-unique, and common m^6^A peaks are shown; **(B)** Venn diagram of m^6^A peak–represented transcripts of the two groups; **(C)** summary of m^6^A-modified genes identified in the m^6^A-seq. **(D)** The top six motifs enriched across the m^6^A peaks identified from HS. **(E–I)** The proportion of m^6^A peak distributions: the proportion of m^6^A peak distribution in the indicated regions in NS and HS samples **(E)**; the proportion of m^6^A peak distribution in the indicated regions in NS **(F)**; the proportion of m^6^A peak distribution in the indicated regions in HS **(G)**; the loss of existing m^6^A peaks (NS-unique peaks) **(H)** or appearance of new m^6^A peaks (HS-unique peaks) **(I)** in the HS group. HS, hypertrophic scar group; NS, normal skin group.

Through the model analysis of the HS group ChIP-seq (MACS) (Zhang et al., 2008), a total of 27,210 m^6^A peaks were identified, representing 9,636 gene transcripts. In the adjacent NS group, 20,254 m^6^A peaks were identified, corresponding to 8,169 gene transcripts (Figures 1A,B). The difference and overlap of m^6^A RNA in the HS and NS groups are shown by the Venn diagrams in Figures 1A,B. Among them, there were a total of 12,419 m^6^A peaks corresponding to 6,973 m^6^A modifier genes in the two groups of samples. Compared with the NS group, 14,791 new peaks appeared in the HS group and 7,835 peaks disappeared, indicating that the overall m^6^A modification patterns of the HS and NS groups were significantly different (Figures 1A–C).

The m^6^A methylomes were further mapped using HOMER software. Among the 35,045 identified m^6^A peaks, the most consistent motifs were GAAGA and GGACU (Figure 1D). We analyzed the distribution of m^6^A in the entire transcriptome of HS and NS samples. The total and unique peaks of the two sets of m^6^A were analyzed. According to the position of the m^6^A peak in the RNA transcript, the m^6^A peak can be divided into the 5′-UTR, start codon segment (400 nucleotides centered on the start codon), coding sequence (CDS), stop codon segment (400 nucleotides centered on the stop codon), and 3′-UTR. In general, the m^6^A peak was particularly enriched near the start codon, CDS, and stop codon (Figure 1G), which was consistent with previous m^6^A-seq results (Dominissini et al., 2012; Meyer et al., 2012). The m^6^A peak of HS was significantly different from that of NS. The deposition of m^6^A in the HS start codon region was relatively increased, and the stop codon region was relatively reduced, whereas it was the opposite in the NS group (Figures 1E–I). The 7,835 unique peaks of NS included 700 peaks in the 5′-UTR region, 1,198 peaks in the start codon, 3,893 peaks in the CDS region, 1,750 peaks in the stop codon, and 294 peaks in the 3′-UTR region (Figure 1G), while in HS, 14,791 m^6^A-specific peaks included 1,556 peaks in the 5′-UTR region, 2,775 peaks in the start codon, 7,838 peaks in the CDS region, 2,290 peaks in the stop codon, and 333 peaks in the 3′-UTR region (Figure 1I).

### Abnormal m^6^A-Modified Genes Were Enriched in Fibrosis-Related Signaling Pathways

**FIGURE 2:**
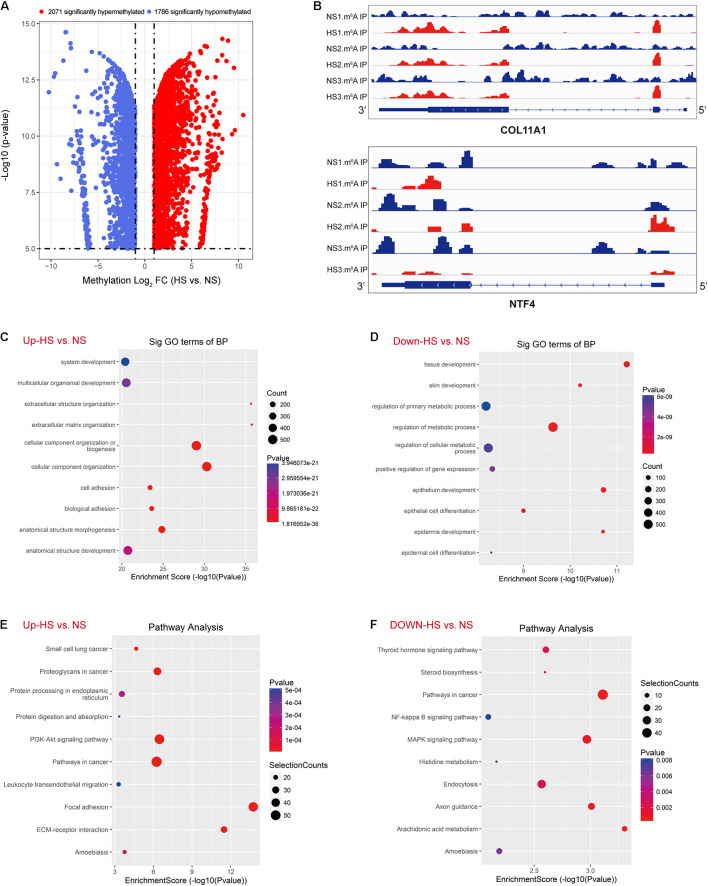
Global m^6^A modification changes in hypertrophic scar (HS) tissues compared with normal skin (NS) tissues. **(A)** Volcano plots displaying the distinct m^6^A peaks (fold change > 2, *p* < 0.05). **(B)** The m^6^A abundances in COL10A1 and NTF4 transcripts in NS and HS samples, as detected by m^6^A-seq. **(C)** Gene ontology (GO) analysis of biological process involved in up-methylated genes in HS samples conducted by top GO. **(D)** GO analysis of the biological process involved in down-methylated genes in HS samples conducted by top GO. **(E)** Gene set enrichment pathway analysis of transcripts with increased m^6^A modification in HS samples compared with NS samples by top GO. **(F)** Pathway analysis of transcripts with reduced m^6^A modification in HS samples compared with NS samples. HS, hypertrophic scar group; NS, normal skin group.

The abundance of m^6^A peaks in the HS and NS samples were compared. In the 35,045 m^6^A peaks detected in the two samples, a total of 10,832 differential methylation sites were used for further study. The top 20 differently methylated m^6^A peaks are listed in Table 3. Compared with the NS group, the HS group had 2,071 significantly hyper-methylated m^6^A sites and 1,786 significantly hypo-methylated m^6^A sites (fold change > 2, *p* < 0.05) (Figure 2A). The remaining m^6^A peaks were regarded as unchanged m^6^A peaks. According to the Integrated Genomics Viewer (IGV) software (Oda et al., 2000), COL11A1 and NTF4, a significantly hypermethylated peak is shown in Figure 2B. The intensity of the differentially methylated sites in the two groups changed, and the GGACU motif surrounded the corresponding m^6^A peak. To determine the biological significance of m^6^A methylation in HS/NS, the GO and KEGG pathways were analyzed for genes (differentially methylated genes, DMGs) with significantly changed m^6^A peaks in HS/NS. GO analysis found that the hypermethylated genes in HS/NS were significantly involved in system development (ontology: biological process Figure 2C), fibrillar collagen trimer (ontology: cellular component Supplementary Figure 1A), and binding (ontology: molecular function; Supplementary Figure 1C). Hypomethylated genes were significantly related to tissue development (ontology: biological process Figure 2D), intracellular part (ontology: Supplementary Figure 1B), and MAP kinase activity (ontology: molecular function; Supplementary Figure 1D) regulation. Of note, the KEGG pathway analysis showed that hypermethylated genes in HS/NS were closely associated with the P13K-Akt signaling pathway, focal adhesion, and ECM-receptor interaction (Figure 2E), while hypomethylated genes in HS/NS were closely related to the MAPK signaling pathway and the NF-kappa B signaling pathway (Figure 2F).

**TABLE 3:** The top 20 differently expressed m6A peaks (SC vs. NS).

Gene name	Fold change	Regulations	Chromosome	Peak region	Peak start	Peak end	*P*-value
COL11A1	1451.023256	Up	chr1	cds	103354132	103354186	1.15401E-11
COL8A1	783.7	Up	chr3	5utr	99463740	99463840	5.43491E-11
ANO4	737.3	Up	chr12	cds	101333092	101333229	9.24614E-14
COL11A1	637.1860465	Up	chr1	cds	103453188	103453296	8.24947E-11
SFRP4	473.1	Up	chr7	cds	37953974	37954055	5.71842E-15
SPP1	421.4	Up	chr4	cds	88902626	88902950	4.59346E-14
LRRC15	314.0691244	Up	chr3	3utr	194077961	194078240	1.33691E-10
TNN	310.1	Up	chr1	cds	175046519	175046780	4.72961E-15
RUNX1	306.5	Up	chr21	5utr	36206061	36206260	2.50702E-14
SALL4	296.9	Up	chr20	cds	50400781	50401060	5.39635E-10
ISL1	1206.4	Down	chr5	3utr	50689501	50689920	1.11391E-12
CYP1A1	826.4	Down	chr15	5utr	75015361	75015467	2.29393E-13
AR	750.5	Down	chrX	cds	66764721	66765080	1.60835E-13
ISL1	660.9	Down	chr5	3utr	50689981	50690320	1.76552E-09
AL353791.1	505.8	Down	chr9	3utr	40032121	40032417	7.83885E-09
PCSK5	410.5	Down	chr9	3utr	78973881	78974440	4.41149E-14
DDX3Y	348.3	Down	chrY	3utr	15031361	15031740	2.4175E-15
MYOCD	338.1	Down	chr17	3utr	12667461	12667900	2.39283E-15
PPARGC1A	266.7	Down	chr4	3utr	23796461	23797020	2.26285E-10
DDX3Y	243.1	Down	chrY	cds	15026475	15026600	7.47452E-15

### Identification of Differentially Expressed Genes in Hypertrophic Scar/Normal Skin by RNA-Seq

**FIGURE 3:**
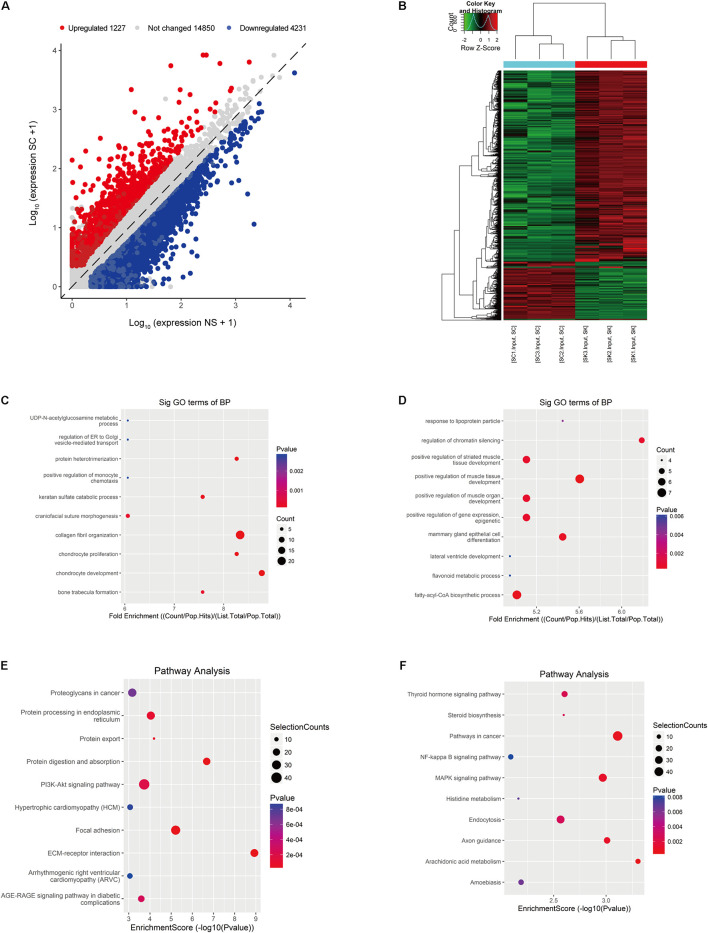
Identification of differentially expressed genes (DEGs) in HS by RNA-seq. **(A)** Scatter plot of the RNA-seq data. **(B)** Heat map of RNA-seq data of HS and NS samples. Rows: mRNAs; columns: normal skin group and hypertrophic scar samples. Red, black, and green indicate the upregulation, unchanged expression, and downregulation of mRNAs, respectively. **(C)** Gene ontology (GO) analysis of the biological process involved in upregulated genes in HS samples conducted by top GO. **(D)** GO analysis of the biological process involved in downregulated genes in HS samples conducted by top GO. **(E)** Gene set enrichment pathway analysis of transcripts with increased mRNA modification in HS samples compared with NS samples by top GO. **(F)** Pathway analysis of transcripts with reduced mRNA modification in HS samples compared with NS samples. HS, hypertrophic scar group; NS, normal skin group.

In the RNA-seq data set (m^6^A-seq input library), we found significant differences in the overall mRNA expression patterns between HS samples and adjacent NS. The results of the mRNA expression profile analysis showed that compared with the control group, 5,458 mRNA expressions were significantly altered in the HS group, of which 1,227 mRNA expressions were upregulated and 4,231 mRNA expressions were downregulated (fold change > 2, *p* < 0.05). The top 20 altered genes are listed in Table 4. Figures 3A,B shows scatter plots and hierarchical clustering of the RNA-seq data. The GO and KEGG pathways were analyzed for differentially expressed genes (DEGs). The results showed that abnormally upregulated genes in the HS/NS samples were related to collagen fibril organization (ontology: biological process Figure 3C), the endomembrane (ontology: Supplementary Figure 2A), and collagen binding (ontology: molecular function; Supplementary Figure 2C). Regulation is closely related. Abnormally downregulated genes were significantly associated with the positive regulation of muscle tissue development (ontology: biological process Figure 3D), cornified envelope (ontology: cellular component Supplementary Figure 2B), and binding (ontology: molecular function; Supplementary Figure 2D). It is of note that the KEGG pathway analysis showed that abnormally upregulated genes in HS/NS were closely associated with the P13K-Akt signaling pathway, focal adhesion, and ECM–receptor interaction (Figure 3E), while the abnormally downregulated genes in HS/NS were closely associated with the MAPK signaling and NF-kappa B signaling pathways (Figure 3F).

**TABLE 4:** The top 20 differently expressed genes (SC vs. NS).

Gene name	Fold change	Regulations	Locus	Strand	*P*-value
COL10A1	2351.719046	Up	chr14:68086514-68162531	−	0.0051
PALM2-AKAP2	1985.793649	Up	chr8:22435791-22461663	+	0.04
ADAM12	253.2660696	Up	chr6:116422011-116570660	−	0.00585
LRRC15	248.5185962	Up	chr9:112403067-112934792	−	0.048
DNAH12	194.6328516	Up	chr10:127700949-128077024	−	0.00005
ASPN	193.5134456	Up	chr3:194075975-194090472	−	0.00005
ALPK2	173.3266879	Up	chr3:57327726-57530071	−	0.00005
IGF2BP3	171.4507019	Up	chr9:95059639-95432547	−	0.00005
COL11A1	140.9744594	Up	chr18:56148478-56296189	−	0.00005
HOXB6	108.5134453	Up	chr18:61616534-61672278	−	0.01735
NTF4	8720624.367	Down	chr22:51007289-51052409	−	0.0407
TUBA8	139838	Down	chr14:24641061-24708448	+	0.01675
SLC25A1	80349.4	Down	chr16:15489610-15737023	−	0.0495
KCNIP2	13497.5	Down	chr6:32116135-32145873	−	0.00085
ANKRD23	5698.24	Down	chr19:11562140-11639989	−	0.0006
MACROD1	513.949	Down	chr19:16589867-16771253	−	0.00075
PLXNB3	495.627	Down	chr2:170335687-170382772	+	0.03
TNFRSF1B	472.362	Down	chr2:53759809-54087297	+	0.01915
KRT4	384.5880166	Down	chr12:53200332-53208335	−	0.00015
ADAMTS19	−353.3122596	Down	chr5:128795251-129522327	+	0.0118

### Conjoint Analysis of m^6^A-RNA Binding Protein Immunoprecipitation (MeRIP)-Seq and RNA-Seq Data of Hypertrophic Scar and Normal Skin

**FIGURE 4:**
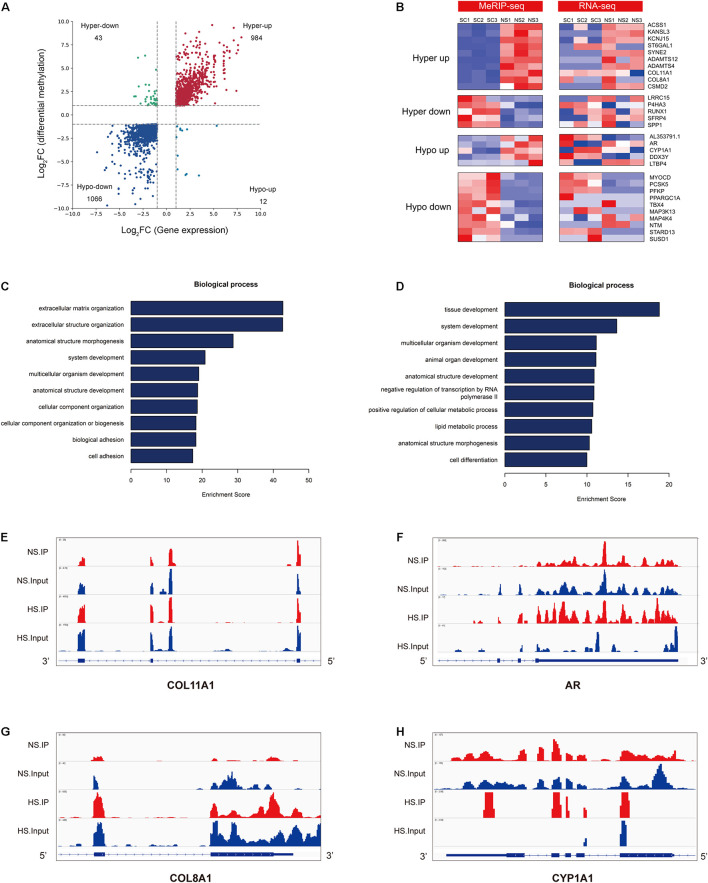
Conjoint analysis of m^6^A-RIP-seq and RNA-seq data. **(A)** Distribution of genes with a significant change in both m^6^A and mRNA levels in hypertrophic scar (HS) samples compared with normal skin (NS) tissues (fold change > 2, *p* < 0.05). **(B)** Heat map of “hyper-up,” “hyper-down,” “hypo-up,” and “hypo-down” genes represented in panel **(A)**. **(C)** The top 10 gene ontology (GO) terms of the biological process for the “hyper-up” genes in HS compared with NS. **(D)** The top 10 GO terms of the biological process for the “hypo-down” genes in HS compared with NS. **(E–H)** Data visualization of the top four differently expressed genes containing differently methylated peaks in Table 5. **(E)** Data visualization of COL11A1 mRNA m^6^A modification in HS compared with NS. **(F)** Data visualization of AR mRNA m^6^A modification in HS compared with NS. **(G)** Data visualization of COL8A1 mRNA m^6^A modification in HS compared with NS. **(H)** Data visualization of CYP1A1 mRNA m^6^A modification in HS compared with NS.

**FIGURE 5:**
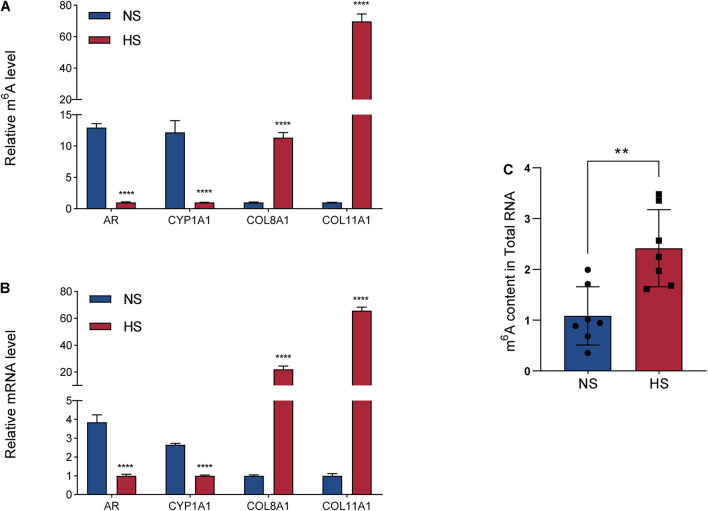
Gene-specific m6A qPCR assays and detection of global m^6^A levels. **(A)** Gene-specific m^6^A qPCR validation of m^6^A level changes of four representative hypermethylated or hypomethylated genes in the normal skin (NS) and hypertrophic scar (HS) groups. **(B)** Relative mRNA levels of four representative genes were measured by real-time PCR in the NS and HS groups. **(C)** m^6^A/A ratio of polyA-RNAs isolated from the NS and HS samples were determined using the m^6^A RNA Methylation Assay Kit. The bar shows the mean from *n* = 7 technical replicates. **P = 0.0030, ****P < 0.0001.

MeRIP-seq was used to detect DMGs between HS and NS (Figure 2A), while RNA-seq was used to detect DEGs between HS and NS (Figure 3A). Through the conjoint analysis of the DMGs and DEGS, all genes were mainly divided into four groups, including 984 hypermethylated and upregulated genes (hyper-up), 1,066 hypomethylated and downregulated genes (hypo-down), 43 hypermethylated but downregulated genes (hyper-down), and 12 hypomethylated but upregulated genes (hypo-up) (Figure 4A). Compared with the normal control, the genes with significant changes in m^6^A levels and mRNA transcription abundance in HS samples are listed in Table 5, and then hierarchical cluster analysis was performed on these genes (Figure 4B). It is of note that 96% (1,066/1,109) of downregulated mRNA transcripts were associated with m^6^A hypomethylation in the HS samples. In addition, the numbers of “hyper-up” and “hypo-down” genes were more than those of the “hyper-down” and “hypo-up” genes, with larger fold changes and smaller *p*-values. These results showed that m^6^A modification was positively correlated with mRNA expression in HS. In the conjoint analysis of DMGs and DEGS, we performed GO enrichment analysis on hyper-up and hypo-down genes. The “hyper-up” genes were associated with ECM organization, extracellular structure organization, and anatomical structure morphogenesis (ontology: biological process Figure 4C), while the “hypo-down” genes were related to tissue development, system development, and multicellular organization development (ontology: biological process Figure 4D). The enrichment analysis of cell components and molecular functions are presented in Supplementary Figure 3. Data visualization of the top four differently expressed genes (COL11A1, COL8A1, CYP1A1, and AR) containing differently methylated peaks are presented in Figures 4E–H. To further confirm the results of our m^6^A-seq data, we conducted gene-specific m^6^A qPCR assays for several hyper-methylated and hypo-methylated genes (COL11A1, COL8A1, and CYP1A1, AR) (Figure 5A). We observed changes in m^6^A levels consistent with the sequencing results in these four genes, which indicated the reliability of our transcriptome-wide m^6^A-seq data. In turn, the mRNA levels of the above genes were detected in three pairs of NS and HS samples (Figure 5B). These results showed that the trend for m^6^A methylation was similar and the mRNA expression was consistent with the sequencing results. Finally, the overall m^6^A level of NS and HS samples was measured using an m^6^A colorimetry kit. HS samples showed higher relative total m^6^A levels than NS samples (Figure 5C). This showed that the HS samples had a unique m^6^A modification pattern that was different from normal tissues, including the transcriptome range and gene-specific scale.

**TABLE 5:** Thirty genes that exhibit a significant change in both m6A and mRNA transcript abundance (SC vs. NS).

Gene name	Pattern	Chromosome	m6A level change					mRNA level change		
			Peak region	Peak start	Peak end	Fold change	*p*-value	Strand	Fold change	*p*-value
COL11A1	Hyper-up	chr1	cds	103354132	103354186	1451.0233	1.154E-11	−	140.9744594	0.00005
COL8A1	Hyper-up	chr3	5utr	99463740	99463840	783.7	5.435E−11	+	28.80625085	0.00005
SFRP4	Hyper-up	chr7	cds	37953974	37954055	473.1	5.718E−15	−	11.03725876	0.00005
SPP1	Hyper-up	chr4	cds	88902626	88902950	421.4	4.593E-14	+	inf	0.00005
LRRC15	Hyper-up	chr3	3utr	194077961	194078240	314.06912	1.337E-10	−	248.5185962	0.00005
RUNX1	Hyper-up	chr21	cds	36206061	36206260	306.5	2.507E-14	−	9.135919889	0.00005
ADAMTS4	Hyper-up	chr1	5utr	161167784	161168520	239	8.66E-11	−	8.741131244	0.0259
ADAMTS12	Hyper-up	chr5	cds	33643501	33643575	230.5	3.329E-10	−	5.901943051	0.00005
CSMD2	Hyper-up	chr1	3utr	34064401	34065140	222.9	3.073E-11	−	11.67588499	0.00005
P4HA3	Hyper-up	chr11	cds	73988029	73988189	9.2097902	3.907E-07	+	−3.62615351	0.00005
KCNJ15	Hyper-down	chr21	cds	39601836	39601945	81.2	7.134E-07	+	−4.86297778	0.00005
ST6GAL1	Hyper-down	chr3	cds	186692744	186692878	28.547619	2.968E-08	+	−2.17491648	0.0123
ACSS1	Hyper-down	chr20	cds	25002024	25002172	15.25	1.539E-07	−	−7.6686892	0.00005
KANSL3	Hyper-down	chr2	cds	97265461	97266120	6.0776699	1.36E-06	−	−2.0923004	0.00005
SYNE2	Hyper-down	chr14	cds	64640616	64640727	20.52381	3.413E-06	−	2.261797861	0.0118
MAP3K13	Hypo-up	chr3	3utr	185206041	185206240	86.2	1.603E-07	+	4.046318825	0.00015
SUSD1	Hypo-up	chr9	cds	114804441	114804445	80	1.075E-06	−	4.375749045	0.01515
MAP4K4	Hypo-up	chr2	cds	102314164	102314180	56.6	6.098E-06	+	2.240296991	0.00015
NTM	Hypo-up	chr11	cds	131240370	131240783	212.2	2.07E-09	−	49.38667718	0.00005
STARD13	Hypo-up	chr13	cds	34235413	34235480	151.7	6.099E-11	+	−2.65880874	0.0004
CYP1A1	Hypo-down	chr15	5utr	75015361	75015467	826.4	2.294E-13	−	−79.6217127	0.00005
AR	Hypo-down	chrX	cds	66764721	66765080	750.5	1.608E-13	+	-10.0674751	0.00005
AL353791.1	Hypo-down	chr9	3utr	40032121	40032417	505.8	7.839E-09	+	−6.85178556	0.02565
PCSK5	Hypo-down	chr9	3utr	78973881	78974440	410.5	4.411E-14	+	−2.75013035	0.0073
DDX3Y	Hypo-down	chrY	3utr	15031361	15031740	348.3	2.418E-15	+	−34.3314765	0.00005
MYOCD	Hypo-down	chr17	3utr	12667461	12667900	338.1	2.393E-15	+	−12.1720478	0.00635
PPARGC1A	Hypo-down	chr4	3utr	23796461	23797020	266.7	2.263E-10	−	−12.0944227	0.00005
LTBP4	Hypo-down	chr19	cds	41107276	41107440	233.5	1.216E-14	+	−4.78725946	0.00005
PFKP	Hypo-down	chr10	cds	3159201	3159780	5.2233062	1.281E-12	+	2.067847796	0.0081
TBX4	Hypo-down	chr17	3utr	59560721	59561320	233.2	1.408E-10	+	−inf	0.00005

## Discussion

Similar to DNA modification, there are a series of reversible modifications on RNA. m^6^A modification is one of the most common RNA modifications. The m6A modification targets and regulates the stability (Wang et al., 2014), localization (Wang et al., 2014), transport, and translation of mRNA after transcription (Wang et al., 2015), thereby affecting various biological processes, including embryonic development, stem cell self-renewal, DNA damage response, and primary miRNA processing (Wang et al., 2014; Weng et al., 2019; Liu et al., 2021). The epigenetic layer of this emerging role in HS has not yet been characterized. In this study, we showed the overall m^6^A modification pattern in HS samples and in adjacent NS tissues. Analysis found that the genes regulated through abnormal m^6^A RNA modification were closely associated with fibrosis-related pathways. In addition, we found that the m^6^A modification pattern in the HS samples was different from that of the normal control. A higher total m^6^A level and more than 6,956 m^6^A peaks were identified in the HS group. In addition, we performed GO and KEGG pathway analyses to infer the potential function of m^6^A-modified transcript changes. Finally, conjoint analysis of m^6^A-RIP-seq and RNA-seq data revealed that m^6^A-modified mRNA transcripts were hypermethylated or hypomethylated, and their expressions are significantly different. Based on the potential role of m^6^A modification in the pathogenesis of HS, reversing the changes in m^6^A levels may be an attractive new treatment strategy. The combined analysis of our m^6^A-seq and mRNA-seq data revealed 30 genes in the HS group that had different methylated m^6^A sites and changes in mRNA abundance compared with the NS group (fold change > 2, *p* < 0.05, Table 5). These genes may play a key role in the occurrence and development of hypertrophic scar, thus these are of interest for further investigation. Through GO enrichment analysis, COL11A1, COL8A1, CYP1A1, and AR were shown to be associated with ECM organization and tissue development. To study polymorphisms of genes encoding enzymes in alcohol-related diseases, an investigation involving 120 Brazilian alcoholics and 221 controls with similar ethnic backgrounds was performed. The results showed that subjects with an m2/m2 CYP1A1 genotype were more likely to have alcoholic liver cirrhosis (Burim et al., 2004). It has been reported that CYP1A1 polymorphism may increase the risk for oral submucosal fibrosis (Chaudhuri et al., 2013). Excessive collagen accumulation is frequently used to assess fibrosis development to determine the collagen gene associated with conjunctival fibrosis after glaucoma filtering surgery (GFS). The study used a mouse model of GFS to identify collagen transcripts through RNA-seq that were upregulated during the fibrotic stage of wound healing. The most increased collagen transcripts were encoded by Col8A1, Col11A1, and Col8A2. They increased 67, 54, and 18 times, respectively, in the fibrosis stage (Seet et al., 2017). Therefore, further functional studies may help to clarify the molecular mechanisms of the aforementioned genes in the occurrence and development of HS. In addition, regulating m^6^A modification may become a strategy for the treatment of human diseases in the future.

In the present study, when depicted on a global scale, m^6^A modification and mRNA expression in HS samples tended to have a positive correlation (Figure 4A). This result was further confirmed by gene-specific m^6^A qPCR and mRNA qPCR analyses (Figures 5A,B). The results of our study proved for the first time the association between m^6^A methylation and HS pathogenesis, and its potential clinical significance for HS patients. In addition, it was reported that m^6^A modification affected the stability, localization, transportation, and translation of the target mRNA. The sample size in this study is limited. Follow-up studies require a larger sample size to study the reasons for the overall m6A change in HS. The specific effect of m^6^A methylation on gene expression largely depends on the modification of methylases, demethylases, and methylation recognition enzymes (Fu et al., 2014; Deng et al., 2018). In addition, functional experiments are needed to further confirm the regulatory role of m6A RNA modifications upon gene expression in HS. To further confirm the regulatory effect of m^6^A RNA modification on gene expression in HS, knocking-out or overexpressing the key enzymes of m^6^A modification may provide a good strategy for studying the cellular response mediated by m^6^A methylation.

## Conclusion

This study presented the first m^6^A full transcriptome map of human HS. It provided a potential link between abnormal m^6^A RNA modification and fibrosis-related gene expression. We hope this may provide a starting point to investigate the features of m^6^A and may offer some guidance for further research on m^6^A modification in HS.

## Data Availability Statement

The original contributions presented in the study are publicly available. This data can be found here: Gene Expression Omnibus GSE181540 at: https://www.ncbi.nlm.nih.gov/geo/query/acc.cgi?acc=GSE181540.

## Ethics Statement

The studies involving human participants were reviewed and approved by The Affiliated Hospital of Jiangnan University. The patients/participants provided their written informed consent to participate in this study.

## Author Contributions

F-LY and S-YL designed and supervised the whole study. S-YL performed the experiments. J-JW and Z-HC provided the statistical analysis and wrote the article. D-YG, K-WZ, M-LZ, Y-YT, and Y-YL collected the samples and clinical data, and revised the article. F-LY gave final approval of the version to be published. All authors contributed to the article and approved the submitted version.

## Conflict of Interest

The authors declare that the research was conducted in the absence of any commercial or financial relationships that could be construed as a potential conflict of interest.

## Publisher’s Note

All claims expressed in this article are solely those of the authors and do not necessarily represent those of their affiliated organizations, or those of the publisher, the editors and the reviewers. Any product that may be evaluated in this article, or claim that may be made by its manufacturer, is not guaranteed or endorsed by the publisher.

## Footnotes

1
https://www.ncbi.nlm.nih.gov/geo/query/acc.cgi?acc=GSE181540

## Supplementary Material

Supplementary Figure 1 Gene ontology enrichment of altered m^6^A transcripts. **(A)** Gene ontology (GO) analysis of the cellular component involved in up-methylated genes in HS samples conducted by top GO. **(B)** GO analysis of the cellular component involved in down-methylated genes in HS samples conducted by top GO. **(C)** GO analysis of the molecular function involved in up-methylated genes in HS samples conducted by top GO. **(D)** GO analysis of the molecular function involved in down-methylated genes in HS samples conducted by top GO.

Supplementary Figure 2 Gene ontology enrichment of altered mRNA transcripts. **(A)** Gene ontology (GO) analysis of the cellular component involved in upregulated genes in HS samples conducted by top GO. **(B)** GO analysis of the cellular component involved in downregulated genes in HS samples conducted by top GO. **(C)** GO analysis of the molecular function involved in upregulated genes in HS samples conducted by top GO. **(D)** GO analysis of the molecular function involved in downregulated genes in HS samples conducted by top GO. HS, hypertrophic scar.

Supplementary Figure 3 Gene ontology enrichment of altered m^6^A and mRNA transcripts in hypertrophic scar samples compared with normal skin tissues. **(A)** Gene ontology analysis of the cellular component involved in “hyper-up” genes in HS samples conducted by top GO. **(B)** GO analysis of the cellular component involved in “hypo-down” genes in HS samples conducted by top GO. **(C)** GO analysis of the molecular function involved in “hyper-up” genes in HS samples conducted by top GO. **(D)** GO analysis of the molecular function involved in “hypo-down” genes in HS samples conducted by top GO. HS, hypertrophic scar.
