# Supplementary figures and images for: The m6A RNA Modification Modulates Gene Expression and Fibrosis-Related Pathways in Hypertrophic Scar
Source: Front Cell Dev Biol. 2021 Nov 15;9:748703. doi: 10.3389/fcell.2021.748703 (PMC8634666; doi:10.3389/fcell.2021.748703)

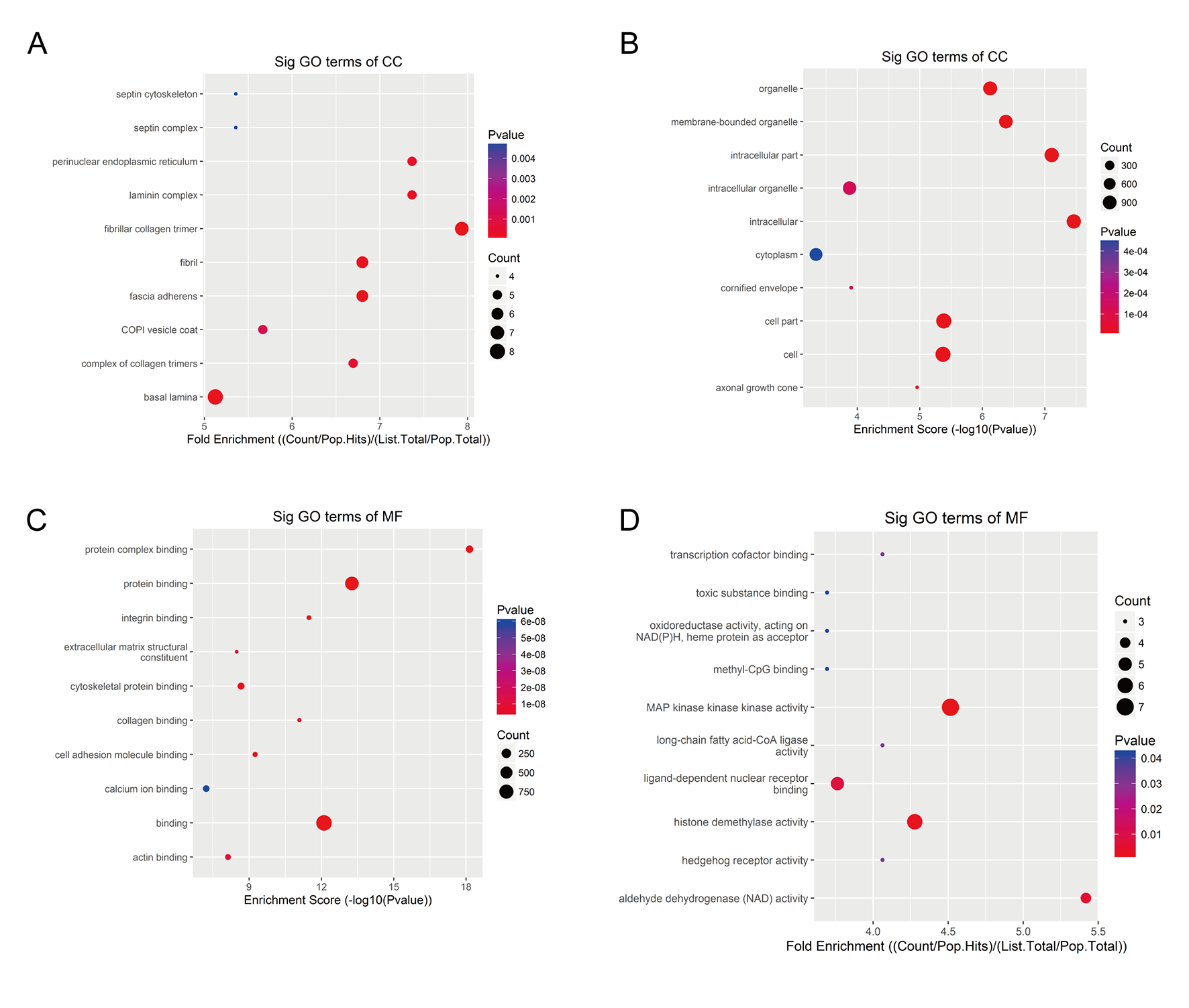

Supplement: Supplementary Figure 1 — Gene ontology enrichment of altered m6A transcripts. (A) Gene ontology (GO) analysis of the cellular component involved in up-methylated genes in HS samples conducted by top GO. (B) GO analysis of the cellular component involved in down-methylated genes in HS samples conducted by top GO. (C) GO analysis of the molecular function involved in up-methylated genes in HS samples conducted by top GO. (D) GO analysis of the molecular function involved in down-methylated genes in HS samples conducted by top GO. [file Image_1.TIF]

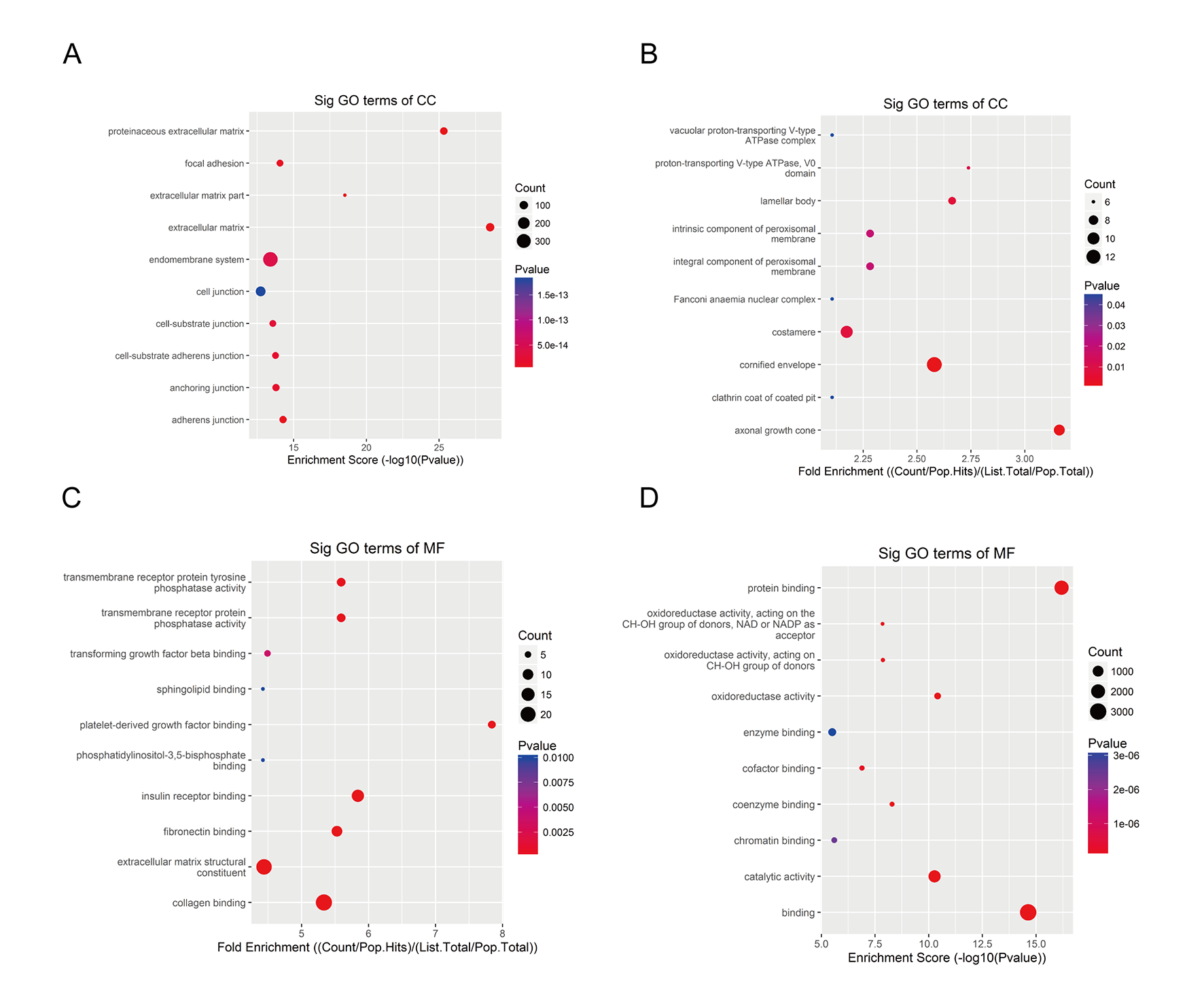

Supplement: Supplementary Figure 2 — Gene ontology enrichment of altered mRNA transcripts. (A) Gene ontology (GO) analysis of the cellular component involved in upregulated genes in HS samples conducted by top GO. (B) GO analysis of the cellular component involved in downregulated genes in HS samples conducted by top GO. (C) GO analysis of the molecular function involved in upregulated genes in HS samples conducted by top GO. (D) GO analysis of the molecular function involved in downregulated genes in HS samples conducted by top GO. HS, hypertrophic scar. [file Image_2.TIF]

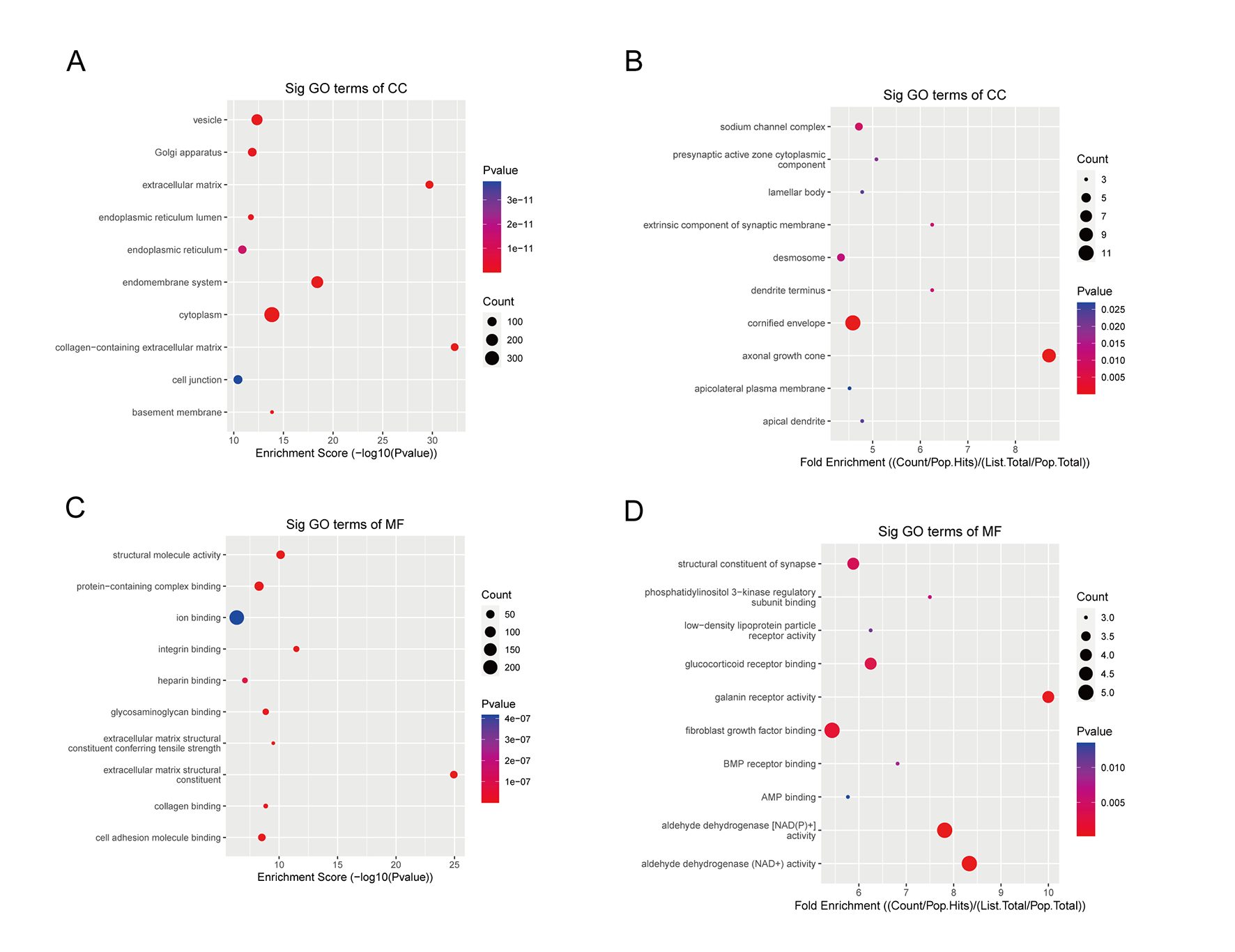

Supplement: Supplementary Figure 3 — Gene ontology enrichment of altered m6A and mRNA transcripts in hypertrophic scar samples compared with normal skin tissues. (A) Gene ontology analysis of the cellular component involved in “hyper-up” genes in HS samples conducted by top GO. (B) GO analysis of the cellular component involved in “hypo-down” genes in HS samples conducted by top GO. (C) GO analysis of the molecular function involved in “hyper-up” genes in HS samples conducted by top GO. (D) GO analysis of the molecular function involved in “hypo-down” genes in HS samples conducted by top GO. HS, hypertrophic scar. [file Image_3.TIF]
